# Recurrent venous thromboembolism: association with thrombin generation and d-dimer

**DOI:** 10.1016/j.rpth.2026.103391

**Published:** 2026-02-13

**Authors:** Eugenia Biguzzi, Kristien Winckers, Tilman M. Hackeng, Frits R. Rosendaal, Astrid van Hylckama Vlieg

**Affiliations:** 1 Department of Clinical Epidemiology, Leiden University Medical Center, Leiden, The Netherlands; 2 Fondazione IRCCS Ca’ Granda Ospedale Maggiore Policlinico, Milan, Italy; 3 Clinic of Hematology, Oncology Institute of Southern Switzerland, Ente Ospedaliero Cantonale, Bellinzona, Switzerland; 4 Department of Biochemistry, Cardiovascular Research Institute Maastricht (CARIM), University Maastricht, The Netherlands

**Keywords:** anticoagulants, blood coagulation tests, pulmonary embolism, venous thromboembolism

## Abstract

**Background:**

In patients with a first event of venous thromboembolism (VTE) clinicians must decide when to stop anticoagulation, evaluating the risk of recurrent thrombosis.

**Objectives:**

The aim of this study was to evaluate the association between recurrent VTE and d-dimer and thrombin generation.

**Methods:**

Analyses were performed in 1895 individuals of the Multiple Environmental and Genetic Assessment of Risk Factors for Venous Thrombosis (MEGA) case-control study, followed up for recurrent VTE. The cumulative incidence of recurrent VTE, incidence rates, and hazard ratios were estimated by Cox proportional hazard regression models, to obtain rates and risks of VTE for different levels of d-dimer and thrombin generation.

**Results:**

Three classes of risk of recurrent VTE were shown: low risk for patients with low d-dimer and thrombin generation (incidence rate, 1.20/100 patient years; 95% confidence interval [CI], 0.75-1.62), intermediate risk for patients with either high d-dimer and low thrombin generation (2.31; 95% CI, 1.97-2.66) or low d-dimer and high thrombin generation (2.89; 95% CI, 0.06-5.73), and high risk for patients with high d-dimer and thrombin generation (4.70; 95% CI, 3.31-6.09).

In the subgroup of patients with unprovoked VTE (*n* = 539), the 3 levels of risk of recurrent VTE were confirmed: low risk in patients with low d-dimer and thrombin generation (incidence rates per 100 patient years, 1.36; 95% CI, 0.42-2.30); intermediate risk in patients with high d-dimer (3.89; 95% CI, 3.07-4.70) and high risk in patients with high d-dimer and thrombin generation (9.80; 95% CI, 5.28-14.33).

**Conclusions:**

The combination of these 2 tests allows identification of patients with high risk of recurrent VTE who could benefit from long-term anticoagulation.

## Essentials

• After venous thromboemboliasm (VTE), clinicians must evaluate the risk of recurrent VTE.
• Risk of recurrent VTE was evaluated for different levels of D-dimer and thrombin generation.
• Risk of recurrent VTE was associated with D-dimer and thrombin generation.
• Unprovoked VTE with low D-dimer and thrombin generation, has low risk of recurrent VTE.

## 1 Introduction

Venous thromboembolism (VTE) has been treated with anticoagulation by heparin since the 1930s. This treatment has evolved with vitamin K antagonists, low molecular weight heparin, and more recently direct oral anticoagulants, but bleeding events can still be a severe complication in anticoagulated patients [1]. Nowadays, this is even more important in the aging population, characterized by a higher incidence of VTE events but also by a higher bleeding risk than young individuals, due to other ongoing treatments (antiplatelet drugs for arterial thrombosis) or comorbidities.

Therefore, clinicians need to evaluate both the risk of recurrent VTE and of bleeding after an initial course of anticoagulation of at least 3 months [1]. Several studies have shown that prolonging anticoagulant treatment is beneficial to prevent recurrent VTE, but the benefit ceases when anticoagulation is stopped [[2], [3], [4], [5]]. For this reason, the alternative is to continue anticoagulation indefinitely after a first event of VTE, after a careful evaluation of risks and benefits. In cases of a first event associated with transient risk factors, such as surgery, immobilization, bone fractures, and the use of oral contraceptives, removing the associated factor lowers the risk of recurrence. In these patients, anticoagulant treatment can be stopped [1]. In case of a first event not associated with transient risk factors and low bleeding risk, indefinite anticoagulation may be considered, especially in case of male patients and pulmonary embolism as first event. High levels of d-dimer, measured 1 month after discontinuation of anticoagulation, have been associated with an increased risk of recurrent VTE [6,7], and the evaluation of d-dimer is currently included in several prediction models for recurrent VTE (DASH, HER-DOO2, and Vienna Prediction Model) [[8], [9], [10], [11], [12], [13], [14], [15]]. Thrombin generation is another tool to evaluate the risk of recurrent VTE, since it measures the ability to produce thrombin in an individual’s plasma [[16], [17], [18], [19]].

Since d-dimer is a degradation product of fibrin, it represents presence of fibrin, while thrombin generation represents the potency of plasma to produce thrombin (and subsequently fibrin) in the presence of a trigger. The aim of the present study was to evaluate the association between these 2 tests (alone or in combination) and recurrent VTE.

## 2 Methods

### 2.1 Data collection and blood collection

The Multiple Environmental and Genetic Assessment of Risk Factors for Venous Thrombosis (MEGA) study is a multicenter, population-based, case-control study on risk factors for VTE (first event of deep vein thrombosis or pulmonary embolism), that enrolled 4956 patients and 6297 controls between 1999 and 2004. Individuals with a history of VTE, severe psychiatric problems, or inability to speak Dutch were excluded. Participants were invited to fill in a questionnaire on risk factors for venous thrombosis and to donate a blood sample (3 months after the discontinuation of anticoagulant therapy). Blood samples, due to logistic reasons, were collected until June 2002 for a total of 2367 patients. Patients included in the MEGA case-control study were followed up for a recurrent event [20]. Between June 2008 and July 2009, patients were sent a questionnaire concerning recurrent VTE. To avoid loss to follow-up, in case of not-returned questionnaires, patients were reached by telephone interview. Information on recurrent events during follow-up was also obtained via anticoagulation clinics where patients initially were treated for their first event. When patients moved house during follow-up, information was retrieved from the clinic nearest to their new address. Vital status of all follow-up patients was acquired from the central Dutch population register between 2007 and 2009. In case of death, causes of death were obtained from the central register of death certificates of the Central Bureau of Statistics.

Recurrence of VTE was determined from discharge letters from the anticoagulant clinics or, in case of death, from registered cause of death [20]. To be classified as a certain recurrent VTE, the event should fulfill one of the following criteria: 1) a discharge letter was present, concluding with a diagnosis of recurrence and including information about instrumental diagnostic procedures; 2) if a discharge letter was not available, both the anticoagulation clinic and the patient had to report a clearly different location from the first event, or a period of more than a year had passed between the 2 events; 3) in the case of death from VTE, at least 6 months had to have passed after the first event. In case of ipsilateral deep vein thrombosis or recurrent pulmonary embolism, the event was classified as a certain recurrence if at least 3 months had passed since the first thrombosis [20].

Duration of follow-up was defined as the time between the date of discontinuation of anticoagulant treatment and end of follow-up, which was defined as the date of a recurrent event or, in the absence of a recurrence, the date that the follow-up questionnaire was completed. When patients did not fill in the questionnaire, they were censored at the last date known to be free of recurrence. This could be date of death, emigration, last visit to anticoagulation clinic or the last time known to be recurrence free from the information provided from the MEGA case-control study.

Patients were excluded when at blood sampling they presented one of the following conditions, since they are potentially associated with significant changes in thrombin generation and d-dimer: 1) active cancer in the last 5 years; 2) use of anticoagulant therapy; and 3) pregnancy or postpartum period. Patients who developed a recurrent event during treatment with oral anticoagulants were also excluded.

All patients gave written informed consent and the study was approved by the Medical Ethics Committee of the Leiden University Medical Center.

### 2.2 Laboratory measurements

Blood samples were collected between 1999 and 2003 collected into tubes containing trisodium citrate 0.106 mmol/L and centrifuged at 2000 *g* for 10 minutes after which plasma was frozen and stored in aliquots at 80 °C. d-dimer and thrombin generation were performed during 2013–2014.

d-dimer was measured using the HemosIL d-dimer HS 500 kit, according to the manufacturer’s instructions. Thrombin generation was measured by calibrated automated thrombography as described by Hemker et al. [21]. To prevent contact activation, contact activation inhibitor thermostable inhibitor of contact activation was added to all plasma samples immediately upon defrosting to a final concentration of 30 μg/mL. Thrombin generation was measured under 2 different assay conditions: low tissue factor concentration (∼2 pM) and high tissue factor concentration (10 pM, with or without activated protein C).

For thrombin generation with low tissue factor, we analyzed the thrombin peak height and endogenous thrombin potential (ETP), which is the area under the curve representing the net amount of thrombin that can be generated. For thrombin generation performed at high tissue factor concentration, measured with and without activated protein C (APC) and subsequently normalized against a normal pool plasma, we analyzed the normalized activated protein C sensitivity ratio (nAPCsr), calculated as follows:

(ETP with APC/ETP without APC)_patients_/(ETP with APC/ETP without APC)_normal pool plasma_ [22].

Plasma samples were collected between 1999 and 2003 and stored at −80°C. d-dimer and thrombin generation were performed in 2013-2014.

All laboratory measurements were performed by staff who had no knowledge of whether the sample was from a patient or a control subject.

### 2.3 Statistical analysis

All statistical analyses were performed with SPSS 27.0 statistical package for Windows, IBM corp.

Results were reported as median values and interquartile range (IQR) for continuous parameters and as number and percentages for categorical variables. Age was assessed at venipuncture. The normal distribution of variables was visually evaluated using histograms.

The risk of venous thrombosis was estimated for deciles of thrombin generation and d-dimer in order to assess at which cut-off values the risk of recurrent venous thrombosis was increased and which subsequently could be used to dichotomize patients into low or high levels of thrombin generation and d-dimer for the combined analysis.

Kaplan–Meier survival curves were plotted to visualize the cumulative incidence of VTE recurrence. The cumulative incidence of recurrent VTE at 2 and 5 years of follow-up was expressed as number of events/100 patients at risk. Crude incidence rates with 95% confidence intervals (CIs) of recurrent VTE were estimated as the number of events over the accumulated follow-up time (per 100 patient years). Cox proportional hazard regression models were used to calculate hazard ratios (HRs) and 95% CI to evaluate relative risks of VTE associated with different levels of thrombin generation parameters and d-dimer. The HRs were adjusted for age, sex, and duration of anticoagulation (time between index date and discontinuation of anticoagulation). The analysis was repeated in subgroups of patients with a first provoked or unprovoked VTE. Unprovoked first VTE was defined as a first event in the absence of hormone use, pregnancy, puerperium, plaster cast, trauma, surgery, immobilization in the last 3 months before the event, and >4 hours traveling in the last 2 months.

In a sensitivity analysis, follow-up started at the date of blood sampling with adjustment for time between the date of discontinuation of anticoagulation therapy and blood sampling.

## 3 Results

**Figure 1:**
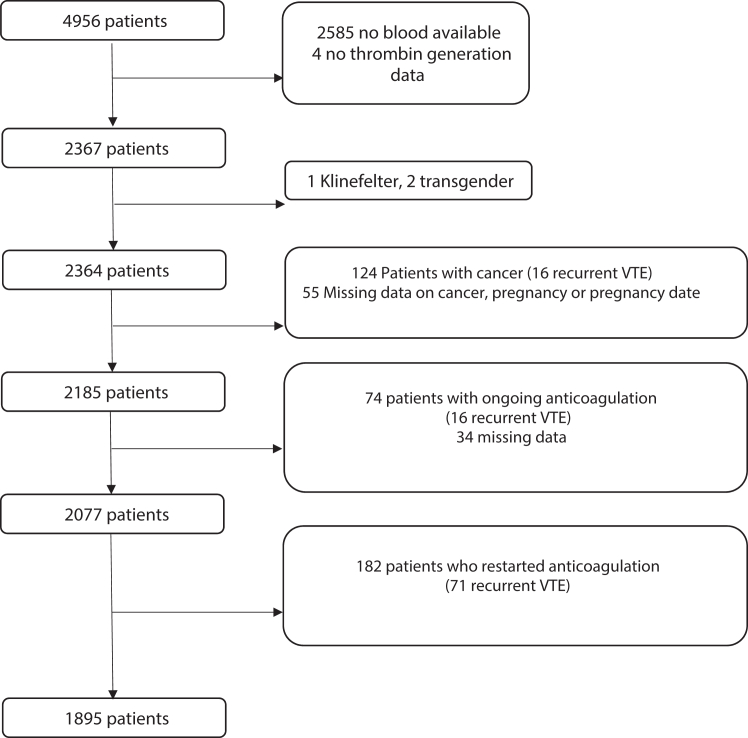
Study desing flowchart. VTE, venous thromboembolism.

After exclusion of 4 patients because thrombin generation data were not available due to technical reasons and 472 patients due to one or more of the exclusion criteria (1 Klinefelter, 2 transgender, 124 patients with cancer, 89 patients with missing data, 74 patients with did had not stopped anticoagulation at the time of blood collection, and 182 patients who had restarted anticoagulation), a total of 1895 patients with a first episode of VTE were observed for a recurrent event (Figure 1). Patient characteristics are shown in Table 1. The median time of follow-up was 7.0 years (IQR, 4.0-9.1 years), and 252 recurrent VTE events were observed in 11,144 person-years, with an overall incidence rate of recurrence of 2.26 per 100 person-years (95% CI, 1.98-2.54) in the whole group. One hundred and one patients with recurrent VTE were excluded from the analysis (16 patients with cancer, 16 patients on oral anticoagulation at the time of blood collection, and 71 patients because they had restarted oral anticoagulation at the time of blood collection).

**Table 1:** Characteristics of the investigated population (patients).

Number of subjects	1895
Sex (men; *N*, %)	845, 45%
Age (y), median (IQR)	50 (39-58)
Presence of risk factors at 1st VTE	
Provoked, (*N*, %)	1329, 71%
Unprovoked, (*N*, %)	539, 29%
Type of thrombosis at 1st VTE	
Isolated deep vein thrombosis, (*N*, %)	1144, 60%
Pulmonary emobolism, (*N*, %)	751, 40%
Duration of anticoagulant treatment (mo), median (IQR)	5.9 (3.5-6.8)
Number of recurrent events, (*N*, %)	252, 13%
Follow-up (y), median (IQR)	7.0 (4.0-8.1)

Continuous variables are shown as median (interquartile range); categorical variables as number (%)

The median value of d-dimer in all patients was 327 ng/mL (IQR, 223-533 ng/mL). The median (and IQR) value of nAPCsr was 2.12 (1.30-3.60).

**Figure 2:**
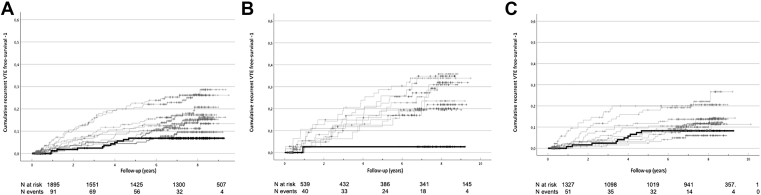
Kaplan–Meier curves: the risk of recurrent VTE during follow-up time is shown for the different deciles of d-dimer. Kaplan–Meier curves of the whole group are shown in panel A, of the unprovoked VTE in panel B and of the provoked VTE group of patients in panel C. The black thick line shows the lowest decile, the gray thin lines depict deciles 2 to 10. Number of patients at risk and number of events for 2-years interval are shown at the bottom of each panel. VTE, venous thromboembolism.

**Figure 3:**
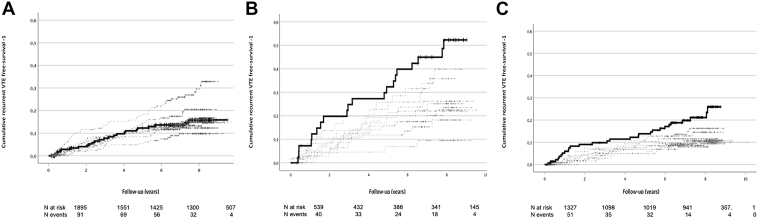
Kaplan–Meier curves: the risk of recurrent VTE during follow-up time is shown for the different deciles of nAPCRsr (high tissue factor concentration). Kaplan–Meier curves of the whole group are shown in panel A, of the unprovoked VTE in panel B and of the provoked VTE group of patients in panel C. The black thick line shows the lowest decile, the gray thin lines depict deciles 2 to 10. Number of patients at risk and number of events for 2-years interval are shown at the bottom of each panel. VTE, venous thromboembolism.

Tables 2 and 3 and Figures 2 and 3 show cumulative incidences at 2 years and 5 years, incidence rates, HRs (crude and adjusted) of recurrent VTE and Kaplan–Meier curves associated with increasing deciles of d-dimer and nAPCsr. The incidence rate increased with d-dimer deciles when compared with the lowest decile of d-dimer. The incidence rate was particularly low in patients with low levels of d-dimer with an unprovoked first VTE (Table 2 and Figure 2).

**Table 2:** d-dimer in patients with and without recurrent venous thromboembolism.

d-dimer (ng/mL) Deciles (cutoff)	Patients *N* (%)	Observation years	Recurrent events	2-Y cumulative incidence (%)	5-Y cumulative incidence (%)	Incidence rate (95% CI)	HR (95% CI)	Adjusted HR^a^ (95% CI)
1st decile (≤161)	189	1165	11	1.7	6.3	0.94 (0.39-1.50)	Reference	Reference
2nd decile (201)	190	1164	19	1.7	6.9	1.63 (0.90-2.37)	1.72 (0.82-3.62)	1.86 (0.85-4.07)
3rd decile (244)	189	1186	24	6.1	9.5	2.02 (1.21-2.83)	2.15 (1.05-4.39)	2.33 (1.10-4.93)
4th decile (281)	190	1093	21	3.5	6.5	1.92 (1.10-2.74)	2.02 (0.98-4.19)	2.67 (1.21-5.87)
5h decile (327)	189	1105	26	4.0	11.0	2.35 (1.45-3.26)	2.51 (1.24-5.08)	3.02 (1.40-6.51)
6th decile (389)	190	1130	14	2.3	4.1	1.24 (0.59-1.89)	1.31 (0.59-2.88)	1.81 (0.77-4.29)
7th decile (475)	190	1181	25	5.6	10.7	2.12 (1.29-2.95)	2.23 (1.10-4.53)	2.78 (1.28-6.05)
8th decile (612)	189	1120	31	6.7	13.2	2.77 (1.79-3.74)	2.89 (1.45-5.75)	3.47 (1.66-7.26)
9th decile (828)	190	1038	42	10.0	21.3	4.05 (2.82-5.27)	4.21 (2.17-8.18)	5.21 (2.48-10.9)
10th decile (>828)	189	962	39	9.9	19.1	4.06 (2.78-5.33)	4.19 (2.14-8.18)	4.93 (2.92-10.59)

Unprovoked								
d-dimer (ng/mL) deciles	Patients *N* (%)	Observation years	Recurrent events	2-Y cumulative incidence (%)	5-Y cumulative incidence (%)	Incidence rate (95% CI)	HR (95% CI)	Adjusted HR^a^ (95% CI)
1st decile	46	292	1	2.4	2.4	0.34 (-0.33-1.01)	Reference	Reference
2nd decile	47	269	8	4.5	13.8	2.98 (0.91-5.04)	7.95 (0.99-63.6)	7.52 (0.90-62.8)
3rd decile	48	293	9	14.7	17.0	3.07 (1.07-5.08)	8.49 (1.07-67.1)	8.55 (1.05-69.5)
4th decile	36	198	8	6.0	18.5	4.04 (1.24-6.84)	11.9 (1.48-95.2)	11.0 (1.32-91.5)
5h decile	45	255	13	4.7	24.1	5.10 (2.33-7.88)	15.3 (1.99-117.1)	24.1 (2.81-206.7)
6th decile	40	221	7	5.6	11.1	3.17 (0.82-5.51)	9.32 (1.14-75.9)	11.02 (1.27-95.9)
7th decile	60	361	11	10.6	14.4	3.04 (1.25-4.84)	8.72 (1.13-67.6)	8.93 (1.03-77.4)
8th decile	59	342	16	7.1	18.2	4.68 (2.39-6.98)	13.7 (1.82-103.6)	23.8 (2.96-191.3)
9th decile	72	375	21	11.9	26.9	5.59 (3.20-7.99)	15.1 (2.03-112.6)	20.8 (2.49-173.5)
10th decile	86	452	21	7.6	20.6	4.65 (2.66-6.64)	13.0 (1.74-96.4)	15.5 (1.91-125.5)

Provoked								
d-dimer (ng/mL) deciles	Patients *N* (%)	Observation years	Recurrent events	2-Y cumulative incidence (%)	5-Y cumulative incidence (%)	Incidence rate (95% CI)	HR (95% CI)	Adjusted HR^a^ (95% CI)
1st decile	141	866	10	1.5	7.7	1.16 (0.44-1.87)	Reference	Reference
2nd decile	142	895	11	0.8	4.6	1.23 (0.50-1.96)	1.07 (0.45-2.52)	1.23 (0.49-3.10)
3rd decile	138	878	15	3.0	6.9	1.71 (0.84-2.57)	1.49 (0.67-3.31)	1.62 (0.70-3.75)
4th decile	151	873	13	2.9	3.7	1.49 (0.68-2.30)	1.29 (0.56-2.94)	2.02 (0.80-5.12)
5h decile	144	850	13	3.7	6.9	1.53 (0.70-2.36)	1.33 (0.58-3.03)	1.88 (0.77-4.58)
6th decile	148	906	7	1.5	2.2	0.77 (0.20-1.34)	0.67 (0.25-1.75)	0.91 (0.32-2.60)
7th decile	128	804	14	3.3	9.2	1.74 (0.83-2.65)	1.51 (0.67-3.40)	2.50 (1.01-6.18)
8th decile	124	738	15	6.8	11.4	2.03 (1.00-3.06)	1.75 (0.79-3.90)	2.15 (0.91-5.06)
9th decile	115	652	20	9.2	17.2	3.07 (1.72-4.41)	2.65 (1.24-5.67)	3.31 (1.43-7.66)
10th decile	98	481	18	12.4	18.7	3.75 (2.02-5.48)	3.17 (1.46-6.88)	3.93 (1.60-9.63)

CI, confidence interval; HR, hazard ratio; nAPCsr, normalized activated protein C sensitivity ratio.

a adjusted for age, sex, duration of anticoagulation, time from index event to stop of anticoagulation.

**Table 3:** nAPCsr distribution in patients with and without recurrent venous thromboembolism (high tissue factor concentration).

nAPCsrdeciles	Patients *N* (%)	Observation years	Recurrent events	2-Y cumulative incidence (%)	5-Y cumulative Incidence (%)	Incidence rate (95% CI)	HR (95% CI)	Adjusted HR^a^ (95% CI)
1st Decile (≤0.7)	189	1096	24	4.9	11.6	2.19 (1.32-3.07)	Reference	Reference
2nd Decile (1.1)	189	1118	25	7.3	12.0	2.24 (1,36-3.11)	1.02 (0.58-1.79)	1.07 (0.61-1.88)
3rd Decile (1.5)	190	1149	22	3.4	8.0	1.91 (1.11-2.71)	0.88 (0.49-1.56)	1.03 (0.57-1.87)
4th Decile (1.8)	189	1141	22	5.7	10.4	1.93 (1.12-2.73)	0.89 (0.50-1.60)	1.19 (0.65-2.16)
5h Decile (2.1)	189	1160	15	2.3	5.2	1.29 (0.64-1.95)	0.59 (0.31-1.13)	0.61 (0.31-1.23)
6th Decile (2.5)	190	1110	19	3.5	9.3	1.71 (0.94-2.48)	0.80 (0.44-1.46)	1.39 (0.71-2.74)
7th Decile (3.1)	189	1116	24	6.2	10.3	2.15 (1.29-3.01)	0.99 (0.56-1.74)	1.40 (0.76-2.59)
8th Decile (4.2)	190	1098	23	5.1	11.0	2.09 (1.24-2.95)	0.96 (0.54-1.70)	1.28 (0.69-2.39)
9th Decile (5.8)	189	1068	30	2.9	12.9	2.81 (1.80-3.81)	1.27 (0.74-2.18)	1.67 (0.93-3.03)
10th Decile (>5.8)	189	1074	48	11.1	17.5	4.47 (3.20-5.73)	2.05 (1.25-3.34)	2.80 (1.63-4.81)

Unprovoked								
nAPCsr Deciles	Patients N (%)	Observation years	Recurrent events	2-Y cumulative incidence (%)	5-Y cumulative incidence (%)	Incidence rate (95% CI)	HR (95% CI)	Adjusted HR^a^ (95% CI)
1st Decile	75	425	13	4.3	14.7	3.06 (1.39-4.72)	Reference	Reference
2nd Decile	76	437	11	8.3	11.4	2.52 (1.02-4.00)	0.83 (0.37-1.85)	0.83 (0.37-1.86)
3rd Decile	63	374	12	6.7	15.3	3.21 (1.39-5.02)	1.04 (0.47-2.28)	1.07 (0.49-2.34)
4th Decile	56	306	12	9.7	19.8	3,92 (1.70-6.14)	1.29 (0.59-2.83)	1.26 (0.57-2.79)
5h Decile	50	323	4	4.3	6.5	1.24 (0.02-2.45)	0.41 (0.13-1.26)	0.41 (0.13-1.26)
6th Decile	43	236	8	7.6	17.7	3.40 (1.04-5.75)	1.12 (0.46-2.70)	1.28 (0.50-3.24)
7th Decile	43	206	12	12.8	23.7	5.82 (2.53-9.11)	1.93 (0.88-4.23)	2.11 (0.95-4.67)
8th Decile	50	303	11	4.2	19.2	3.63 (1.48-5.77)	1.16 (0.52-2.60)	1.24 (0.55-2.81)
9th Decile	40	216	12	5.3	24.0	5.55 (2.41-8.68)	1.78 (0.81-3.91)	1.75 (0.79-3.87)
10th Decile	43	230	20	19.3	31.3	8.71 (4.89-12.52)	2.81 (1.40-5.65)	2.96 (1.45-6.05)

Provoked								
nAPCsr Deciles	Patients N (%)	Observation years	Recurrent events	2-Y cumulative incidence (%)	5-Y cumulative incidence (%)	Incidence rate (95% CI)	HR (95% CI)	Adjusted HR^a^ (95% CI)
1st Decile	109	646	11	3.9	10.0	1.70 (0.70-2.71)	Reference	Reference
2nd Decile	107	643	13	7.0	12.1	2.02 (0.92-3.12)	1.20 (0.54-2.69)	1.45 (0.65-3.25)
3rd Decile	120	741	10	1.8	4.6	1.35 (0.51-2.19)	0.80 (0.34-1.89)	1.14 (0.46-2.86)
4th Decile	133	836	10	4.0	6.5	1.20 (0.45-1.94)	0.72 (0.31-1.70)	1.16 (0.48-2.82)
5h Decile	136	819	11	1.6	4.8	1.34 (0.55-2.14)	0.79 (0.34-1.82)	0.92 (0.36-2.38)
6th Decile	146	874	11	2.2	6.8	1.26 (0.51-2.00)	0.77 (0.33-1.77)	1.55 (0.58-4.13)
7th Decile	144	901	12	4.4	7.0	1.33 (0.58-2.08)	0.81 (0.36-1.83)	1.06 (0.40-2.83)
8th Decile	139	789	12	5.4	8.1	1.52 (0.66-2.38)	0.90 (0.40-2.04)	1.29 (0.49-3.40)
9th Decile	147	837	18	2.2	9.9	2.15 (1.16-3.14)	1.27 (0.60-2.68)	1.85 (0.77-4.47)
10th Decile	146	845	28	8.7	13.3	3.31 (2.09-4.54)	1.94 (0.97-3.90)	2.76 (1.19-6.42)

CI, confidence interval; HR, hazard ratio; nAPCsr, normalized activated protein C sensitivity ratio.

a Adjusted for age, sex, duration of anticoagulation, time from index event to stop of anticoagulation.

The results obtained using the d-dimer detection limit as cut-off (215 ng/mL) are shown in Supplementary Table 1 and Figure 1. Thrombin peak and ETP obtained with low tissue factor concentration were also associated with an increased risk of recurrent VTE for individuals with levels in the higher deciles compared with those with levels in the lowest decile (shown in Supplementary Tables 2 and 3 and Supplementary Figures 2 and 3).

For the combined analysis of d-dimer and thrombin generation, we used the nAPCsr parameter, which is more sensitive to genetic polymorphisms involved in the activated protein C pathway, such as factor (F)V Leiden, as well as to high levels of FVIII. In addition, nAPCsr is clinically validated through its association with a first event of VTE and also with multiple hereditary and acquired risk factors for venous thrombosis [22,23].

For the combined analysis of d-dimer and thrombin generation, we chose the cut-off of 215 ng/mL for d-dimer, and the highest decile for nAPCsr. The cut-off of 215 ng/mL for d-dimer was chosen because it is the detection limit of the test, and results below this cut-off cannot be differentiated.

The combined analysis in the whole group of patients identified 3 classes of risk of recurrent VTE: low risk for patients with low d-dimer and low thrombin generation (incidence rate, 1.20/100 patient years; 95% CI, 0.75-1.62), intermediate risk for patients with either high d-dimer and low thrombin generation (incidence rate, 2.31/100 patient years; 95% CI, 1.97-2.66) or low d-dimer and high thrombin generation (incidence rate 2.89/100 patient years; 95% CI, 0.06-5.73), and high risk for patients with high d-dimer and high thrombin generation (incidence rate, 4.70/100 patient years; 95% CI, 3.31-6.09).

**Figure 4:**
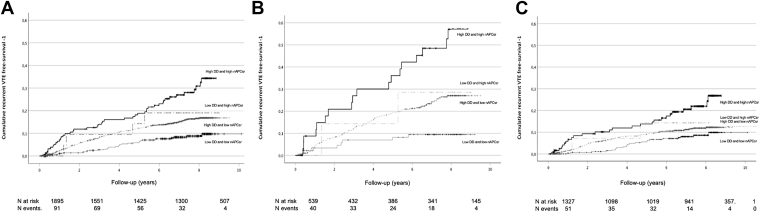
Kaplan–Meier curves: the risk of recurrent VTE during follow-up time is shown for 4 different groups, based on the levels of d-dimer and nAPCsr: low d-dimer (<215 ng/mL) and low nAPCsr (<90° centile) (thin black line); high d-dimer (>215ng/mL) and low nAPCsr (<90° centile) (gray dotted line, - - - -); low d-dimer (<215 ng/mL) and high nAPCsr (>90° centile) (gray dotted line, - - · - -); high d-dimer (>215 ng/mL) and high nAPCsr (>90° centile) (thick black line). Kaplan–Meier curves of the whole group are shown in panel A, of the unprovoked VTE in panel B and of the provoked VTE group of patients in panel C. Number of patients at risk and number of events for 2-years interval are shown at the bottom of each panel. VTE, venous thromboembolism.

In the subgroup of patients with a first event of unprovoked VTE, the combined analysis confirmed 3 levels of risk of recurrent VTE: low risk in patients with low d-dimer and low thrombin generation (incidence rates per 100 patient years 1.36, 95% CI, 0.42-2.30); intermediate risk in patients with high d-dimer (3.89; 95% CI, 3.07-4.70) and high risk in patients with high d-dimer and high thrombin generation (9.80; 95% CI, 5.28-14.33). Only 7 patients presented with isolated high thrombin generation and seem to have an intermediate risk of recurrent VTE, but the CIs are too wide to draw firm conclusions. Table 4 and Figure 4 show results of the combined analysis for the whole group of patients and for the unprovoked and provoked groups.

**Table 4:** Relative risk of recurrent venous throboembolism, according to categories of d-dimer and thrombin generation (nAPCsr, low tissue factor concentration).

d-dimer^a^	nAPCsr^a^	Number of patients	Observation years	Recurrent events	2-Y cumulative incidence (%)	5-Y cumulative incidence (%)	Incidence rate (95% CI)	HR (95% CI)	Adjusted HR (95%)^b^
Low	Low	415	2580	31	1.3	5.8	1.20 (0.78-1.62)	Reference	Reference
High	Low	1289	7478	173	5.5	11.5	2.31 (1.97-2.66)	1.92 (1.31-2.81)	2.00 (1.35-2.97)
Low	High	23	138	4	9.1	13.6	2.89 (0.06-5.73)	2.38 (0.84-6.74)	2.29 (0.80-6.58)
High	High	166	936	44	11.4	18.1	4.70 (3.31-6.09)	3.91 (2.47-6.18)	5.18 (3.18-8.44)

Unprovoked									
d-dimer	nAPCsr	Number of patients	Observation years	Recurrent events	2-Y cumulative incidence (%)	5-Y cumulative incidence (%)	Incidence rate (95% CI)	HR (95% CI)	Adjusted HR (95%)^b^
Low	Low	97	590	8	3.3	7.8	1.36 (0.42-2.30)	Reference	Reference
High	Low	399	2238	87	7.8	18.3	3.89 (3.07-4.70)	2.85 (1.38-5.87)	2.97 (1.43-6.21)
Low	High	7	46	2	14.3	14.3	4.33 (-1.67-10.34)	3.22 (0.68-15.17)	3.06 (0.60-15.63)
High	High	36	184	18	20.3	34.8	9.80 (5.28-14.33)	6.96 (3.02-16.02)	8.59 (3.43-21.50)

Provoked									
d-dimer	nAPCsr	Number of patients	Observation years	Recurrent events	2-Y cumulative incidence (%)	5-Y cumulative incidence (%)	Incidence rate (95% CI)	HR (95% CI)	Adjusted HR (95%)^b^
Low	Low	314	1982	23	0.7	5.2	1.16 (0.69-1.64)	Reference	Reference
High	Low	867	5105	85	4.6	8.5	1.67 (1.31-2.02)	1.43 (0.90-2.27)	1.64 (1.02-2.65)
Low	High	16	92	2	6.7	13.3	2.17 (-0.84-5.18)	1.88 (0.44-7.98)	1.98 (0.46-8.42)
High	High	130	753	26	8.9	13.3	3.46 (2.13-4.78)	2.97 (1.69-5.20)	4.33 (2.34-8.03)

CI, confidence interval; HR, hazard ratio; nAPCsr, normalized activated protein C sensitivity ratio; TF, tissue factor; VTE, venous thromboembolism.

a d-dimer cut-off: low ≤ 215, high >215; nAPCsr cut-off low <90° centile, high ≥90° centile.

b Adjusted for age, sex, duration of anticoagulation, time from index event to stop of anticoagulation.

Results of the sensitivity analysis, where follow-up was started at the date of blood sampling with adjustment for time between the date of discontinuation of anticoagulation therapy and blood sampling, were similar to the results reported for the main analysis and are shown in Supplementary Tables 4–6.

## 4 Discussion

In case of patients with a first event of unprovoked VTE, clinicians need to reassess anticoagulation after 3 months, to decide whether it should be continued indefinitively [1]. This can be a difficult choice in clinical practice, since anticoagulant treatment reduces the risk of recurrent VTE by >80%, but at least part of this benefit is outweighed by the risk of major bleeding events. Moreover, the risk of recurrent VTE is mainly postponed until discontinuation of anticoagulant treatment [[2], [3], [4], [5]].

In the present study the aim was to evaluate the risk of recurrent VTE associated with levels of d-dimer and thrombin generation, after a first event of VTE. Our hypothesis was that high levels of thrombin generation and d-dimer were positively associated with the risk of recurrent VTE, since the use of both tests can give us a picture not only of the present production of fibrin through its degradation products (d-dimer) but also of the potential ability to produce thrombin (thrombin generation). We found that this test combination allows the identification of patients with a high risk of recurrent VTE after a first event of unprovoked VTE. Indeed patients with high d-dimer and high thrombin generation had a higher incidence rate of recurrent VTE per 100 patient years (9.80; 95% CI, 5.28-14.33), compared with patients with low d-dimer and low thrombin generation (1.36; 95% CI, 0.42-2.30), and a higher HR of recurrent VTE (adjusted HR, 8.59; 95% CI, 3.43-21.50).

It has been shown that in patients with a first unprovoked event of VTE, high d-dimer, measured 1 month after discontinuation of anticoagulation, is associated with an increased risk of recurrent VTE [6,7,[24], [25], [26]]. The PROLONG II study also showed the usefulness of repeated d-dimer testing after anticoagulation suspension, to decide a possible therapy resumption [27]. Several prediction models for recurrent VTE after first unprovoked VTE are currently available; the DASH, the HERDOO2, and the Vienna Prediction Model include d-dimer in association with different patient characteristics (age, sex, body mass index, postthrombotic syndrome, hormone use) [[8], [9], [10], [11], [12], [13], [14], [15]].

When we analyzed the incidence of recurrent VTE, we found an association between increasing deciles of d-dimer and thrombin generation and incidence of recurrent VTE.

In patients with a first event of unprovoked VTE, 3 levels of risk of recurrent VTE were identified in the combined analysis of d-dimer and nAPCsr: 1 small group (18% of the total) with low d-dimer and low thrombin generation characterized by a low risk of recurrent VTE; a large group (74%) with high d-dimer associated with a medium risk of recurrent VTE and a small group (7%) with high d-dimer and thrombin generation that shows the highest risk of recurrency.

This distinction could be clinically useful since it allows to identify a group of patients with a first unprovoked VTE where stopping the anticoagulation could be considered safe, since their risk of recurrence of VTE is similar to that of patients with a first event of provoked VTE. Indeed, in this group, the bleeding risk of anticoagulation would outweigh the risk of recurrent VTE. Nevertheless, the majority of patients remain in the intermediate group, where the decision to carry on indefinite anticoagulation must be accurately weighted against the bleeding risk [28].

Our results agree with Eichinger et al. [29], who found an HR of 2.8 (95% CI, 1.5-5.3) of recurrent VTE in patients with both high d-dimer and thrombin generation (reference group with low d-dimer and thrombin generation), after adjustment for age, sex, duration of anticoagulation, FV Leiden, prothrombin G20210A. We chose not to adjust for thrombophilia, since this could be an explanation for high levels of thrombin generation and d-dimer.

Recently the dichotomization of VTE in provoked and unprovoked was criticized [30], and the need of a more precise risk stratification was advocated, to better select patients who may benefit from prolonged anticoagulation. Our study evaluated thrombin generation and d-dimer as biomarkers that might be used in the personalized evaluation of recurrent VTE risk.

The main strength of our study is the large number of patients with VTE. The main limitation is the use of a homemade thrombin generation test, performed in a highly specialized laboratory. This could reduce the translational value of this study since most laboratories may not be able to perform the test routinely. Moreover, the standardization of the thrombin generation assay requires special attention and the International Society on Thrombosis and Haemostasis-Scientific and Standardization Committee recommendations for measuring thrombin generation were recently published to reduce the interlaboratory variability [31]. Thrombin generation is also characterized by interindividual variability with wide reference intervals [32]; however, good stability of thrombin generation parameters over time has been shown in healthy individuals [33,34].

Another limitation of our study is the lack of age-specific d-dimer cut-offs: it was shown in the PROLONG study that for older patients the use of a specific cut-off was necessary to find an association between high d-dimer and risk of recurrent VTE [31,35]. Finally, patients in our study received vitamin K antagonists as anticoagulants, although direct oral anticoagulants are now more commonly prescribed after VTE. d-dimer use in patients taking apixaban did not replicate the results obtained with vitamin K antagonists, possibly because of differences in d-dimer assays or the impact of the COVID-19 pandemic during the trial [36].

In conclusion, this study shows an association between recurrent VTE in patients with unprovoked VTE and high levels of thrombin generation and d-dimer. The use of these 2 tests could aid clinicians in choosing which patients could benefit from indefinite anticoagulation.
