# Supplementary material for: Recurrent venous thromboembolism: association with thrombin generation and d-dimer
Source: Res Pract Thromb Haemost. 2026 Feb 13;10(2):103391. doi: 10.1016/j.rpth.2026.103391 (PMC13067114; doi:10.1016/j.rpth.2026.103391)
Supplement: Supplementary Data [file mmc1.doc]

**SUPPLEMENTARY FIGURES AND TABLES.**

**Supplementary table 1** – D-dimer levels in patients with and without recurrent VTE.

| **D-dimer (ng/mL)** | **Patients**  **N (%)** | **Observation**  **years** | **Recurrent**  **events** | **2-years**  **cumulative**  **Incidence (%)** | **5-years**  **cumulative**  **Incidence (%)** | **Incidence**  **Rate**  **(95% CI)** | **Crude HR**  **(95% CI)** | **Adjusted**  **HR***  **(95% CI)** | **Sensitivity**  **analysis**** |
| --- | --- | --- | --- | --- | --- | --- | --- | --- | --- |
| <=215 ng/ml | 438 | 2718.41 | 35 | 1.7 | 6.2 | 1.29 (0.86-1.71) | Reference | Reference | Reference |
| >215 ng/ml | 1457 | 8425.96 | 217 | 6.2 | 12.3 | 2.58 (2.23-2.92) | 1.99 (1.39-2.85) | 2.15 (1.49-3.11) | 1.91 (1.33-2.73) |
| **UNPROVOKED** |  |  |  |  |  |  |  |  |  |
| **D-dimer (ng/mL)** | **Patients**  **N (%)** | **Observation**  **years** | **Recurrent**  **events** | **2-years**  **cumulative**  **Incidence (%)** | **5-years**  **cumulative**  **Incidence (%)** | **Incidence**  **Rate**  **(95% CI)** | **HR**  **(95% CI)** | **Adjusted**  **HR***  **(95% CI)** | **Sensitivity**  **analysis**** |
| <=215 ng/ml | 104 | 636.06 | 10 | 4.1 | 8.3 | 1.57 (0.60-2.55) | Reference | Reference | Reference |
| >215 ng/ml | 435 | 2421.25 | 105 | 8.8 | 19.7 | 4.34 (3.51-5.17) | 2.74 (1.43-5.24) | 2.90 (1.50-5.62) | 2.62 (1.37-5.02) |
| **PROVOKED** |  |  |  |  |  |  |  |  |  |
| **D-dimer (ng/mL)** | **Patients**  **N (%)** | **Observation**  **years** | **Recurrent**  **events** | **2-years**  **cumulative**  **Incidence (%)** | **5-years**  **cumulative**  **Incidence (%)** | **Incidence**  **Rate**  **(95% CI)** | **HR**  **(95% CI)** | **Adjusted**  **HR***  **(95% CI)** | **Sensitivity**  **analysis**** |
| <=215 ng/ml | 330 | 2073.66 | 25 | 1.0 | 5.6 | 1.21 (0.73-1.68) | Reference | Reference | Reference |
| >215 ng/ml | 999 | 5869.41 | 111 | 5.2 | 9.1 | 1.89 (1.54-2.24) | 1.56 (1.01-2.41) | 1.85 (1.19-2.90) | 1.53 (1.01-2.40) |

*adjusted for age, sex, duration of anticoagulation, time from index event to stop of anticoagulation; ** sensitivity analysis: follow-up started at the date of blood sampling with adjustment

for time between the date of discontinuation of anticoagulation therapy and blood sampling

**Supplementary figure 1** – D-dimer levels in patients with and without recurrent VTE. Kaplan-Meier curves of the whole group are shown in panel A, of the unprovoked VTE in panel B and of the provoked VTE group of patients in panel C. The green line shows patients with d-dimer >215ng/ml, the light blue line patients with d-dimer <=215 ng/ml. Number of patients at risk and number of events for 2-years interval are shown at the bottom of each panel.

**Supplementary table 2–– Thrombin peak distribution in patients with and without recurrent VTE.**

| **Thrombin peak (nM)*** | **Patients**  **N (%)** | **Observation**  **years** | **Recurrent**  **events** | **2-years cumulative**  **Incidence (%)** | **5-years cumulative**  **Incidence (%)** | **Incidence**  **Rate (95% CI)** | **Crude HR**  **(95% CI)** | **Adjusted HR****  **(95% CI)** | **Sensitivity**  **Analysis***** |
| --- | --- | --- | --- | --- | --- | --- | --- | --- | --- |
| 1^st^ decile: <=21 | 189 | 1151.30 | 17 | 4.4 | 7.9 | 1.48 (0.77-2.18) | Reference | Reference | Reference |
| 2^nd^ decile: 26 | 190 | 1126.72 | 18 | 4.5 | 7.4 | 1.60 (0.86-2.34) | 1.08 (0.55-2.09) | 1.02 (0.53-2.00) | 1.10 (0.56-2.15) |
| 3^rd^ decile: 30 | 189 | 1072.80 | 28 | 5.7 | 11.1 | 2.61 (1.64-3.58) | 1.75 (0.96-3.20) | 1.75 (0.95-3.21) | 1.69 (0.91-3.16) |
| 4^th^ decile: 35 | 190 | 1119.49 | 26 | 3.4 | 11.0 | 2.32 (1.43-3.22) | 1.56 (0.86-2.92) | 1.56 (0.85-2.92) | 1.54 (0.81-2.91) |
| 5^h^ decile: 39 | 189 | 1055.13 | 31 | 5.2 | 13.5 | 2.94 (1.90-3.97) | 2.01 (1.11-3.63) | 2.08 (1.14-3.78) | 2.05 (1.12-3.74) |
| 6^th^ decile: 44 | 189 | 1130.32 | 23 | 5.1 | 9.9 | 2.03 (1.20-2.87) | 1.40 (0.75-2.62) | 1.58 (0.84-3.00) | 1.36 (0.71-2.59) |
| 7^th^ decile: 49 | 191 | 1133.91 | 30 | 7.9 | 11.9 | 2.65 (1.70-3.59) | 1.83 (1.00-3.31) | 1.98 (1.09-3.61) | 1.81 (0.98-3.33) |
| 8^th^ decile: 58 | 189 | 1136.61 | 22 | 6.3 | 10.3 | 1.94 (1.13-2.74) | 1.36 (0.72-2.56) | 1.59 (0.84-3.02) | 1.35 (0.70-2.59) |
| 9^th^ decile: 71 | 190 | 1122.14 | 28 | 5.1 | 10.4 | 2.50 (1.57-3.42) | 1.69 (0.93-3.09) | 1.87 (1.00-3.49) | 1.74 (0.94-3.24) |
| 10^th^ decile: >71 | 189 | 1095.95 | 29 | 4.0 | 15.0 | 2.65 (1.68-3.61) | 1.69 (0.93-3.09) | 1.87 (1.00-3.49) | 1.74 (0.94-3.24) |
| **UNPROVOKED** |  |  |  |  |  |  |  |  |  |
| **Thrombin peak (nM)*** | **Patients**  **N (%)** | **Observation**  **years** | **Recurrent**  **events** | **2-years cumulative**  **Incidence (%)** | **5-years cumulative**  **Incidence (%)** | **Incidence**  **Rate (95% CI)** | **Crude HR***  **(95% CI)** | **Adjusted HR****  **(95% CI)** | **Sensitivity**  **Analysis***** |
| 1^st^ decile | 60 | 358.53 | 6 | 7.0 | 8.9 | 1.67 (0.33-3.01) | Reference | Reference | Reference |
| 2^nd^ decile | 68 | 381.96 | 13 | 9.4 | 17.3 | 3.40 (1.55-5.25) | 2.00 (0.76-5.26) | 1.79 (0.67-4.78) | 1.99 (0.75-5.23) |
| 3^rd^ decile | 60 | 292.37 | 15 | 11.0 | 18.7 | 5.13 (2.53-7.73) | 2.94 (1.14-7.59) | 2.93 (1.11-7.75) | 3.18 (1.21-8.37) |
| 4^th^ decile | 61 | 363.47 | 13 | 8.6 | 17.4 | 3.58 (1.63-5.52) | 2.17 (0.82-5.71) | 2.32 (0.85-6.33) | 1.99 (0.74-5.37) |
| 5^h^ decile | 65 | 371.27 | 17 | 6.4 | 23.0 | 4.58 (2.40-6.76) | 2.71 (1.07-6.87) | 2.62 /1.00-6.83) | 2.59 (1.02-6.61) |
| 6^th^ decile | 59 | 320.91 | 11 | 5.6 | 15.4 | 3.43 (1.40-5.45) | 2.06 (0.76-5.58) | 1.80 (0.62-5.29) | 2.01 (0.74-5.49) |
| 7^th^ decile | 45 | 276.35 | 10 | 11.6 | 18.6 | 3.62 (1.38-5.86) | 2.20 (0.80-6.07) | 2.21 (0.78-6.25) | 2.03 (0.72-5.76) |
| 8^th^ decile | 54 | 318.57 | 8 | 6.1 | 12.2 | 2.51 (0.77-4.25) | 1.52 (0.52-4.38) | 1.52 (0.51-4.58) | 1.48 (0.50-4.35) |
| 9^th^ decile | 41 | 232.38 | 15 | 7.7 | 20.8 | 6.45 (3.19-9.72) | 3.82 (1.48-9.84) | 3.85 (1.49-9.96) | 3.78 (1.42-10.1) |
| 10^th^ decile | 26 | 141.19 | 7 | 4.1 | 29.8 | 4.96 (1.29-8.63) | 3.82 (1.48-9.84) | 3.85 (1.49-9.96) | 3.78 (1.42-10.1) |
| **PROVOKED** |  |  |  |  |  |  |  |  |  |
| **Thrombin peak**  **(nM)*** | **Patients**  **N (%)** | **Observation**  **years** | **Recurrent**  **events** | **2-years cumulative**  **Incidence (%)** | **5-years cumulative**  **Incidence (%)** | **Incidence**  **Rate (95% CI)** | **Crude HR**  **(95% CI)** | **Adjusted HR****  **(95% CI)** | **Sensitivity**  **Analysis***** |
| 1^st^ decile | 125 | 771.04 | 11 | 3.3 | 7.7 | 1.43 (0.58-2.27) | Reference | Reference | Reference |
| 2^nd^ decile | 118 | 727.60 | 5 | 1.8 | 1.8 | 0.69 (0.08-1.29) | 0.48 (0.17-1.38) | 0.48 (0.17-1.40) | 0.49 (0.17-1.44) |
| 3^rd^ decile | 127 | 773.14 | 13 | 3.3 | 7.8 | 1.68 (0.77-2.60) | 1.19 (0.53-2.66) | 1.24 (0.55-2.78) | 1.06 (0.45-2.49) |
| 4^th^ decile | 127 | 740.54 | 13 | 0.9 | 7.9 | 1.76 (0.80-2.71) | 1.25 (0.56-2.78) | 1.31 (0.58-2.96) | 1.25 (0.53-2.90) |
| 5^h^ decile | 121 | 673.51 | 14 | 4.5 | 8.4 | 2.08 (0.99-3.17) | 1.47 (0.67-3.23) | 1.79 (0.78-4.11) | 1.59 (0.71-3.59) |
| 6^th^ decile | 126 | 785.51 | 12 | 5.0 | 7.7 | 1.53 (0.66-2.39) | 1.09 (0.48-2.47) | 1.38 (0.59-3.20) | 1.04 (0.44-2.45) |
| 7^th^ decile | 143 | 841.81 | 20 | 6.8 | 10.0 | 2.38 (1.33-3.42) | 1.70 (0.82-3.56) | 2.05 (0.97-4.36) | 1.76 (0.83-3.77) |
| 8^th^ decile | 134 | 809.85 | 14 | 6.4 | 9.7 | 1.73 (0.82-2.63) | 1.27 (0.58-2.80) | 1.64 (0.73-3.72) | 1.27 (0.56-2.90) |
| 9^th^ decile | 147 | 878.87 | 12 | 4.4 | 6.8 | 1.37 (0.59-2.14) | 0.98 (0.43-2.21) | 0.98 (0.40-2.36) | 1.13 (0.48-2.64) |
| 10^th^ decile | 161 | 941.21 | 22 | 4.0 | 12.9 | 2.34 (1.36-3.31) | 0.98 (0.43-2.21) | 0.98 (0.96-1.03) | 1.13 (0.48-2.64) |

* adjusted for age, sex, duration of anticoagulation, time from index event to stop of anticoagulation; *** sensitivity analysis: follow-up started at the date of blood sampling with adjustment for time between the date of discontinuation of anticoagulation therapy and blood sampling

**Supplementary figure 2.** Kaplan-Meier curves: the risk of recurrent VTE during follow-up time is shown for the different deciles of thrombin generation peak. Kaplan-Meier curves of the whole group are shown in panel A, of the unprovoked VTE in panel B and of the provoked VTE group of patients in panel C. The turquoise thick line shows the highest decile. Number of patients at risk and number of events for 2-years interval are shown at the bottom of each panel.

**
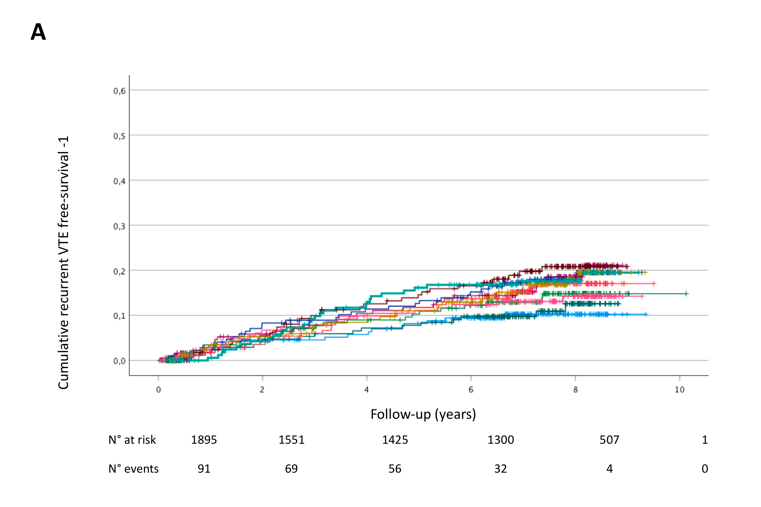

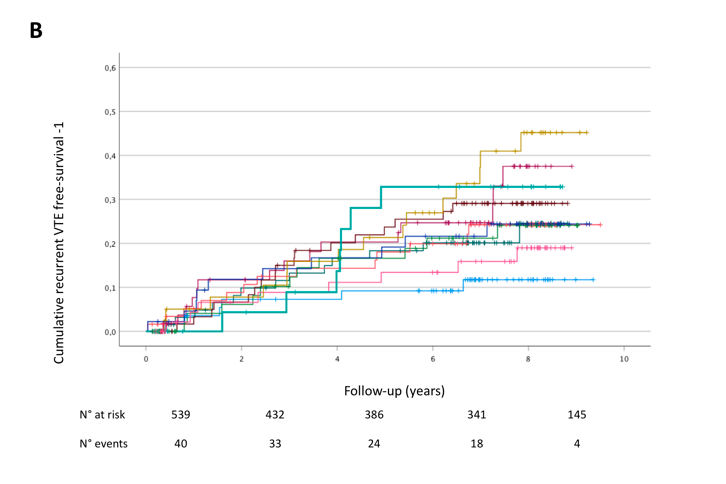

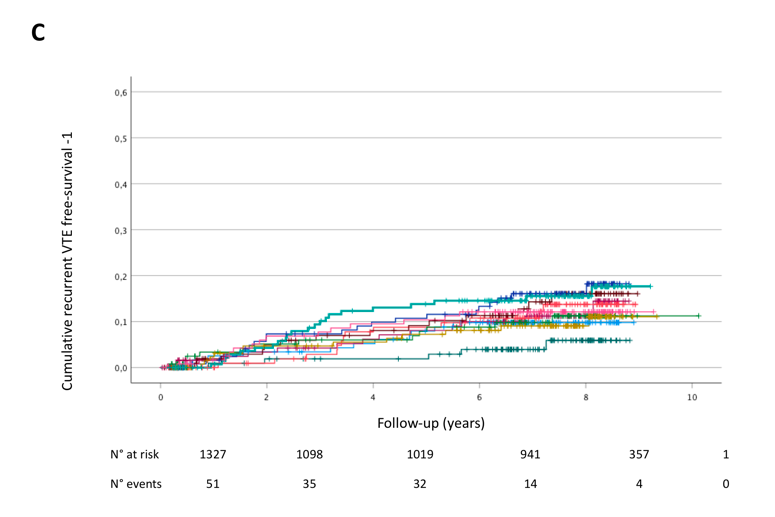
**

**Supplementary table 3 – ETP distribution in patients with and without recurrent VTE**

| **ETP**  **(nM/min)*** | **Patients**  **N** | **Observation**  **years** | **Recurrent**  **events** | **2-years cumulative**  **Incidence (%)** | **5-years cumulative**  **incidence (%)** | **Incidence**  **Rate (95% CI)** | **Crude**  **HR (95% CI)** | **Adjusted**  **HR** (95% CI)** | **Sensitivity**  **Analysis***(95% CI)** |
| --- | --- | --- | --- | --- | --- | --- | --- | --- | --- |
| 1^st^ decile: <=251 | 189 | 1114.45 | 21 | 6.1 | 10.8 | 1.88 (1.08-2.69) | Reference | Reference | Reference |
| 2^nd^ decile: 301 | 190 | 1140.41 | 21 | 2.8 | 6.3 | 1.84 (1.05-2.63) | 0.97 (0.53-1.77) | 0.94 (0.51-1.73) | 0.99 (0.54-1.84) |
| 3^rd^ decile: 336 | 189 | 1109.78 | 31 | 4.5 | 11.6 | 2.79 (1.81-3.78) | 1.48 (0.85-2.58) | 1.52 (0.87-2.65) | 1.47 (0.84-2.60) |
| 4^th^ decile: 367 | 190 | 1102.26 | 20 | 3.4 | 8.8 | 1.81 (1.02-2.61) | 0.97 (0.52-1.79) | 1.02 (0.54-1.92) | 0.96 (0.51-1.80) |
| 5^h^ decile: 395 | 189 | 1016.39 | 31 | 5.8 | 13.1 | 3.05 (1.98-4.12) | 1.64 (0.94-2.85) | 1.80 (1.03-3.14) | 1.63 (0.92-2.87) |
| 6^th^ decile: 427 | 190 | 1163.75 | 22 | 5.6 | 9.7 | 1.89 (1.10-2.68) | 1.03 (0.57- 1.88) | 1.22 (0.67-2.24) | 1.00 (0.54- 1.85) |
| 7^th^ decile: 463 | 190 | 1180.13 | 21 | 5.0 | 7.9 | 1.78 (1.02-2.54) | 0.96 (0.53-1.76) | 1.06 (0.57- 1.97) | 0.89 (0.48-1.67) |
| 8^th^ decile: 510 | 189 | 1100.66 | 24 | 6.9 | 11.1 | 2.18 (1.31-3.05) | 1.18 (0.66-2.14) | 1.46 (0.80-2.68) | 1.16 (0.64-2.12) |
| 9^th^ decile: 581 | 190 | 1136.69 | 26 | 5.6 | 12.0 | 2.29 (1.41-3.17) | 1.21 (0.68-2.16) | 1.59 (0.88-2.87) | 1.19 (0.66-2.14) |
| 10th decile: >581 | 189 | 1079.85 | 35 | 5.7 | 17.3 | 3.24 (2.17-4.31) | 1.73 (1.00-2.98) | 1.79 (0.97-3.31) | 1.82 (1.05-3.15) |
| **UNPROVOKED** |  |  |  |  |  |  |  |  |  |
| **ETP**  **(nM/min)*** | **Patients**  **N** | **Observation**  **years** | **Recurrent**  **events** | **2-years cumulative**  **Incidence (%)** | **5-years cumulative**  **Incidence (%)** | **Incidence**  **Rate (95% CI)** | **Crude**  **HR (95% CI)** | **Adjusted**  **HR** (95% CI)** | **Sensitivity**  **Analysis***(95% CI)** |
| 1^st^ decile | 74 | 419.92 | 12 | 8.6 | 14.6 | 2.86 (1.24-4.47) | Reference | Reference | Reference |
| 2^nd^ decile | 68 | 407.46 | 11 | 6.1 | 10.9 | 2.70 (1.10-4.30) | 0.93 (0.41-2.12) | 0.82 (0.35-1.92) | 0.92 (0.40-2.08) |
| 3^rd^ decile | 69 | 413.69 | 13 | 4.6 | 14.6 | 3.14 (1.43-4.85) | 1.10 (0.50-2.42) | 1.01 (0.45-2.27) | 1.16 (0.52-2.59) |
| 4^th^ decile | 46 | 228.98 | 10 | 9.5 | 19.8 | 4.37 (1.66-7.07) | 1,51 (0.65-3.49) | 1.26 (0.52-3.02) | 1,46 (0.63-3.40) |
| 5^h^ decile | 62 | 346.81 | 16 | 8.5 | 20.7 | 4.61 (2.35-6.87) | 1.61 (0.76-3.41) | 1.62 (0.74-3.55) | 1.45 (0.67-3.11) |
| 6^th^ decile | 54 | 280.44 | 11 | 12.2 | 20.6 | 3.92 (1.60-6.24) | 1.38 (0.61-3.13) | 1.26 (0.55-2.89) | 1.34 (0.58-3.09) |
| 7^th^ decile | 56 | 333.46 | 12 | 7.8 | 11.7 | 3.60 (1.56-5.63) | 1.26 (0.56-2.81) | 1.27 (0.55-2.93) | 1.13 (0.49-2.58) |
| 8^th^ decile | 36 | 216.16 | 7 | 6.0 | 17.9 | 3.24 (0.84-5.64) | 1.16 (0.45-2.96) | 1.19 (0.46-3.06) | 0.99 (0.37-2.66) |
| 9^th^ decile | 39 | 232.50 | 13 | 10.5 | 28.9 | 5.59 (2.55-8.63) | 1.96 (0.89-4.30) | 1.64 (0.72-3.70) | 1.84 (0.83-4.09) |
| 10th decile | 35 | 177.89 | 10 | 6.3 | 26.2 | 5.62 (2.14-9.11) | 1.90 (0.82-4.42) | 1.89 (0.98-4.41) | 1.96 (0.84-4.56) |
| **PROVOKED** |  |  |  |  |  |  |  |  |  |
| **ETP**  **(nM/min)*** | **Patients**  **N** | **Observation**  **years** | **Recurrent**  **events** | **2-years cumulative**  **Incidence (%)** | **5-years cumulative**  **Incidence (%)** | **Incidence**  **Rate (95% CI)** | **Crude**  **HR (95% CI)** | **Adjusted**  **HR** (95% CI)** | **Sensitivity**  **Analysis***(95% CI)** |
| 1^st^ decile | 112 | 678.22 | 9 | 4.6 | 8.6 | 1.33 (0.46-2.19) | Reference | Reference | Reference |
| 2^nd^ decile | 116 | 710.09 | 10 | 0.9 | 3.8 | 1.41 (0.54-2.28) | 1.09 (0.43-2.59) | 1.21 (0.48-3.02) | 1.16 (0.46-2.96) |
| 3^rd^ decile | 116 | 672.60 | 18 | 4.6 | 10.5 | 2.68 (1.44-3.91) | 2.02 (0.91-4.49) | 2.22 (0.99-4.95) | 2.09 (0.90-4.83) |
| 4^th^ decile | 143 | 866.97 | 10 | 1.5 | 5.4 | 1.15 (0.44-1.87) | 0.88 (0.36-2.16) | 0.98 (0.38-2.48) | 0.89 (0.34-2.32) |
| 5^h^ decile | 126 | 662.67 | 15 | 4.5 | 9.2 | 2.26 (1.12-3.41) | 1.76 (0.77-4.02) | 2.40 (1.03-5.58) | 1.95 (0.82-4.64) |
| 6^th^ decile | 133 | 866.71 | 11 | 3.2 | 5.6 | 1.27 (0.52-2.02) | 1.01 (0.42-2.43) | 1.41 (0.57-3.48) | 0.99 (0.32-2.26) |
| 7^th^ decile | 131 | 822.05 | 9 | 4.0 | 6.5 | 1.09 (0.38-1.81) | 0.86 (0.34-2.17) | 1.06 (0.40-2.81) | 0.85 (0.34-2.17) |
| 8^th^ decile | 151 | 880.08 | 16 | 7.1 | 8.8 | 1.82 (0.93-2.71) | 1.43 (0.63-3.23) | 2.20 (0.93-5.18) | 1.55 (0.67-3.63) |
| 9^th^ decile | 149 | 890.73 | 13 | 4.3 | 7.4 | 1.46 (0.67-2.25) | 1.08 (0.46-2.54) | 1.55 (0.60-4.04) | 1.08 (0.44-2.67) |
| 10th decile | 152 | 892.96 | 25 | 5.6 | 15.5 | 2.80 (1.70-3.90) | 2.16 (1.01-4.64) | 1.62 (0.65-4.04) | 2.44 (1.09-5.43) |

* adjusted for age, sex, duration of anticoagulation, time from index event to stop of anticoagulation; *** sensitivity analysis: follow-up started at the date of blood sampling with adjustment for time between the date of discontinuation of anticoagulation therapy and blood sampling

**Supplementary figure 3.** Kaplan-Meier curves: the risk of recurrent VTE during follow-up time is shown for the different deciles of thrombin generation ETP. Kaplan-Meier curves of the whole group are shown in panel A, of the unprovoked VTE in panel B and of the provoked VTE group of patients in panel C. The turquoise thick line shows the highest decile. Number of patients at risk and number of events for 2-years interval are shown at the bottom of each panel.

**
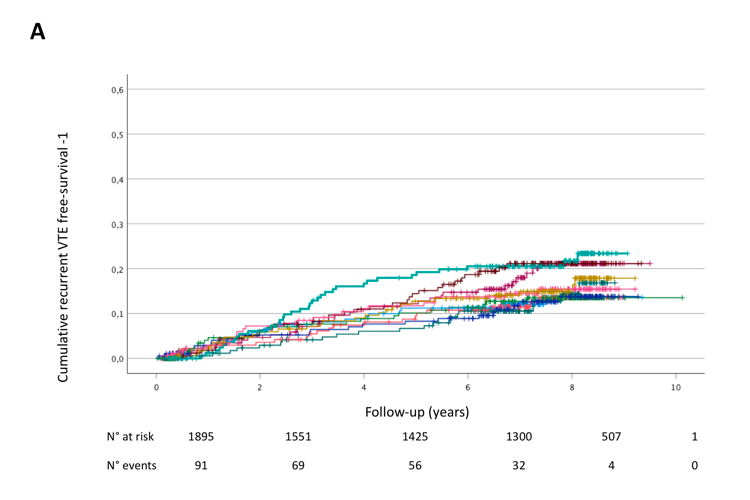

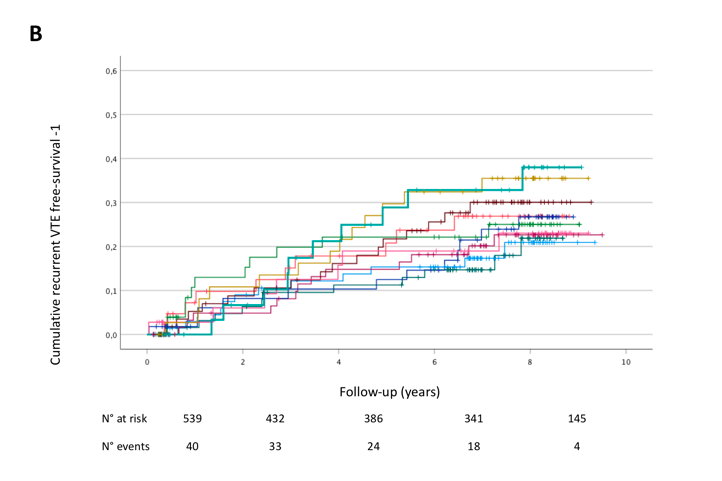

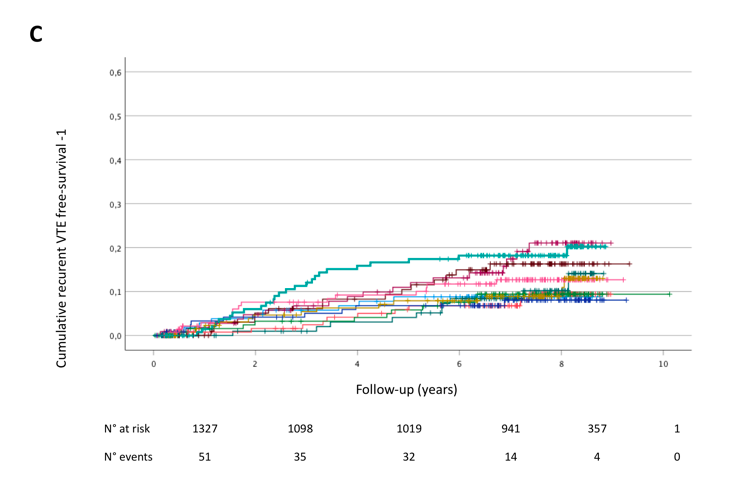
**

**Supplementary table 4** – D-dimer levels in patients with and without recurrent VTE: sensitivity analysis.

| **D-dimer (ng/mL)**  **Deciles*** | **Patients**  **N (%)** | **Observation**  **years** | **Recurrent**  **events** | **HR**  **(95% CI)** | **Sensitivity**  **Analysis**** |
| --- | --- | --- | --- | --- | --- |
| 1^st^ decile: <=161 | 189 | 1165.30 | 11 | Reference | Reference |
| 2^nd^ decile: 201 | 190 | 1163.83 | 19 | 1.72 (0.82-3.62) | 1.74 (0.83-3.65) |
| 3^rd^ decile: 244 | 189 | 1185.68 | 24 | 2.15 (1.05-4.39) | 2.03 (0.99-4.17) |
| 4^th^ decile: 281 | 190 | 1093.26 | 21 | 2.02 (0.98-4.19) | 1.75 (0.83-3.70) |
| 5^h^ decile:327 | 189 | 1104.83 | 26 | 2.51 (1.24-5.08) | 2.52 (1.24-5.12) |
| 6^th^ decile: 389 | 190 | 1130.35 | 14 | 1.31 (0.59-2.88) | 1.32 (0.60-2.90) |
| 7^th^ decile: 475 | 190 | 1181.38 | 25 | 2.23 (1.10-4.53) | 2.14 (1.05-4.37) |
| 8^th^ decile:612 | 189 | 1120.03 | 31 | 2.89 (1.45-5.75) | 2.82 (1.41-5.63) |
| 9^th^ decile:828 | 190 | 1038.07 | 42 | 4.21 (2.17-8.18) | 4.19 (2.16-8.14) |
| 10^th^ decile: >828 | 189 | 961.64 | 39 | 4.19 (2.14-8.18) | 4.09 (2.07-8.06) |
| **UNPROVOKED** |  |  |  |  |  |
| **D-dimer (ng/mL)*** | **Patients**  **N (%)** | **Observation**  **years** | **Recurrent**  **events** | **Crude HR**  **(95% CI)** | **Sensitivity**  **Analysis**** |
| 1^st^ decile | 46 | 292.30 | 1 | Reference | Reference |
| 2^nd^ decile | 47 | 268.54 | 8 | 7.95 (0.99-63.6) | 7.39 (0.92-59.3) |
| 3^rd^ decile | 48 | 292.75 | 9 | 8.49 (1.07-67.1) | 7.47 (0.93-60.1) |
| 4^th^ decile | 36 | 197.91 | 8 | 11.9 (1.48-95.2) | 10.8 (1.32-88.3) |
| 5^h^ decile | 45 | 254.79 | 13 | 15.3 (1.99-117.1) | 13.6 (1.78-105) |
| 6^th^ decile | 40 | 221.09 | 7 | 9.32 (1.14-75.9) | 10.6 (1.26-89.3) |
| 7^th^ decile | 60 | 361.36 | 11 | 8.72 (1.13-67.6) | 7.98 (1.03-61.9) |
| 8^th^ decile | 59 | 341.64 | 16 | 13.7 (1.82-103.6) | 16.9 (2.0-142.5) |
| 9^th^ decile | 72 | 375.35 | 21 | 15.1 (2.03-112.6) | 14,6 (1.96-109) |
| 10^th^ decile | 86 | 451.60 | 21 | 13.0 (1.74-96.4) | 13.5 (1.77-103) |
| **PROVOKED** |  |  |  |  |  |
| **D-dimer (ng/mL)*** | **Patients**  **N (%)** | **Observation**  **years** | **Recurrent**  **events** | **Crude HR**  **(95% CI)** | **Sensitivity**  **Analysis**** |
| 1^st^ decile | 141 | 865.67 | 10 | Reference | Reference |
| 2^nd^ decile | 142 | 894.98 | 11 | 1.07 (0.45-2.52) | 1.08 (0.46-2.55) |
| 3^rd^ decile | 138 | 877.59 | 15 | 1.49 (0.67-3.31) | 1.50 (0.67-3.33) |
| 4^th^ decile | 151 | 872.88 | 13 | 1.29 (0.56-2.94) | 1.09 (0.46-2.58) |
| 5^h^ decile | 144 | 850.04 | 13 | 1.33 (0.58-3.03) | 1.31 (0.56-3.04) |
| 6^th^ decile | 148 | 906.31 | 7 | 0.67 (0.25-1.75) | 0.67 (0.26-1.76) |
| 7^th^ decile | 128 | 804.45 | 14 | 1.51 (0.67-3.40) | 1.43 (0.63-3.25) |
| 8^th^ decile | 124 | 738.23 | 15 | 1.75 (0.79-3.90) | 1.78 (0.80-3.96) |
| 9^th^ decile | 115 | 652.41 | 20 | 2.65 (1.24-5.67) | 2.63 (1.23-5.63) |
| 10^th^ decile | 98 | 480.51 | 18 | 3.17 (1.46-6.88) | 2.87 (1.30-6.34) |

* sensitivity analysis: follow-up started at the date of blood sampling with adjustment for time between the date of discontinuation of anticoagulation therapy and blood sampling

**Supplementary table 5 – nAPCsr distribution in patients with and without recurrent VTE: sensitivity analysis.**

| **nAPCsr**  **deciles** | **Patients**  **N (%)** | **Observation**  **years** | **Recurrent**  **events** | **Incidence**  **Rate (95% CI)** | **Crude HR**  **(95% CI)** | **Sensitivity**  **Analysis**** |
| --- | --- | --- | --- | --- | --- | --- |
| 1^st^ decile:<= 0.7 | 189 | 1096.15 | 24 | 2.19 (1.32-3.07) | Reference | Reference |
| 2^nd^ decile: 1.1 | 189 | 1117.51 | 25 | 2.24 (1,36-3.11) | 1.02 (0.58-1.79) | 1.05 (0.59-1.84) |
| 3^rd^ decile: 1.5 | 190 | 1149.22 | 22 | 1.91 (1.11-2.71) | 0.88 (0.49-1.56) | 0.90 (0.50-1.62) |
| 4^th^ decile: 1.8 | 189 | 1141.47 | 22 | 1.93 (1.12-2.73) | 0.89 (0.50-1.60) | 0.88 (0.49-1.59) |
| 5^h^ decile: 2.1 | 189 | 1160.44 | 15 | 1.29 (0.64-1.95) | 0.59 (0.31-1.13) | 0.53 (0.27-1.05) |
| 6^th^ decile: 2.5 | 190 | 1110.01 | 19 | 1.71 (0.94-2.48) | 0.80 (0.44-1.46) | 0.77 (0.42-1.43) |
| 7^th^ decile: 3.1 | 189 | 1116.67 | 24 | 2.15 (1.29-3.01) | 0.99 (0.56-1.74) | 1.04 (0.58-1.84) |
| 8^th^ decile: 4.2 | 190 | 1098.06 | 23 | 2.09 (1.24-2.95) | 0.96 (0.54-1.70) | 0.94 (0.52-1.68) |
| 9^th^ decile: 5.8 | 189 | 1068.26 | 30 | 2.81 (1.80-3.81) | 1.27 (0.74-2.18) | 1.27 (0.74-2.20) |
| 10^th^ decile:> 5.8 | 189 | 1074.39 | 48 | 4.47 (3.20-5.73) | 2.05 (1.25-3.34) | 2.03 (1.23-3.35) |
| **UNPROVOKED** |  |  |  |  |  |  |
| **nAPCsr**  **deciles** | **Patients**  **N (%)** | **Observation**  **years** | **Recurrent**  **events** | **Incidence**  **Rate (95% CI)** | **Crude HR**  **(95% CI)** | **Sensitivity**  **Analysis**** |
| 1^st^ decile | 75 | 425.34 | 13 | 3.06 (1.39-4.72) | Reference | Reference |
| 2^nd^ decile | 76 | 437.07 | 11 | 2.52 (1.02-4.00) | 0.83 (0.37-1.85) | 0.77 (0.34-1.72) |
| 3^rd^ decile | 63 | 374.13 | 12 | 3.21 (1.39-5.02) | 1.04 (0.47-2.28) | 0.99 (0.45-2.18) |
| 4^th^ decile | 56 | 305.82 | 12 | 3,92 (1.70-6.14) | 1.29 (0.59-2.83) | 1.11 (0.50-2.49) |
| 5^h^ decile | 50 | 323.46 | 4 | 1.24 (0.02-2.45) | 0.41 (0.13-1.26) | 0.38 (0.12-1.18) |
| 6^th^ decile | 43 | 235.62 | 8 | 3.40 (1.04-5.75) | 1.12 (0.46-2.70) | 0.84 (0.33-2.13) |
| 7^th^ decile | 43 | 206.34 | 12 | 5.82 (2.53-9.11) | 1.93 (0.88-4.23) | 2.09 (0.95-4.62) |
| 8^th^ decile | 50 | 303.42 | 11 | 3.63 (1.48-5.77) | 1.16 (0.52-2.60) | 1.01 (0.44-2.31) |
| 9^th^ decile | 40 | 216.37 | 12 | 5.55 (2.41-8.68) | 1.78 (0.81-3.91) | 1.70 (0.76-3.80) |
| 10^th^ decile | 43 | 229.74 | 20 | 8.71 (4.89-12.52) | 2.81 (1.40-5.65) | 2.72 (1.35-5.48) |
| **PROVOKED** |  |  |  |  |  |  |
| **nAPCsr**  **deciles** | **Patients**  **N (%)** | **Observation**  **years** | **Recurrent**  **events** | **Incidence**  **Rate (95% CI)** | **Crude HR**  **(95% CI)** | **Sensitivity**  **Analysis**** |
| 1^st^ decile | 109 | 646.24 | 11 | 1.70 (0.70-2.71) | Reference | Reference |
| 2^nd^ decile | 107 | 643.45 | 13 | 2.02 (0.92-3.12) | 1.20 (0.54-2.69) | 1.32 (0.58-3.02) |
| 3^rd^ decile | 120 | 740.93 | 10 | 1.35 (0.51-2.19) | 0.80 (0.34-1.89) | 0.85 (0.35-2.05) |
| 4^th^ decile | 133 | 835.65 | 10 | 1.20 (0.45-1.94) | 0.72 (0.31-1.70) | 0.77 (0.32-1.85) |
| 5^h^ decile | 136 | 818.71 | 11 | 1.34 (0.55-2.14) | 0.79 (0.34-1.82) | 0.72 (0.29-1.77) |
| 6^th^ decile | 146 | 874.07 | 11 | 1.26 (0.51-2.00) | 0.77 (0.33-1.77) | 0.87 (0.37-2.05) |
| 7^th^ decile | 144 | 901.33 | 12 | 1.33 (0.58-2.08) | 0.81 (0.36-1.83) | 0.86 (0.37-2.01) |
| 8^th^ decile | 139 | 789.06 | 12 | 1.52 (0.66-2.38) | 0.90 (0.40-2.04) | 0.96 (0.42-2.22) |
| 9^th^ decile | 147 | 836.80 | 18 | 2.15 (1.16-3.14) | 1.27 (0.60-2.68) | 1.29 (0.59-2.62) |
| 10^th^ decile | 146 | 844.65 | 28 | 3.31 (2.09-4.54) | 1.94 (0.97-3.90) | 1.97 (0.95-4.09) |

* sensitivity analysis: follow-up started at the date of blood sampling with adjustment for time between the date of discontinuation of anticoagulation therapy and blood sampling

**Supplementary table 6 – Relative risk of recurrent VTE, according to categories of d-dimer and thrombin generation: sensitivity analysis.**

| **D-dimer*** | **nAPCsr*** | **N° patients** | **Observation**  **years** | **N° recurrent VTE** | **Crude HR**  **(95% CI)** | **Sensitivity**  **Analysis**** |
| --- | --- | --- | --- | --- | --- | --- |
| *Low* | *Low* | 415 | 2580.10 | 31 | Reference | Reference |
| *High* | *Low* | 1289 | 7477.68 | 173 | 1.92 (1.31-2.81) | 1.84 (1.25-2.70) |
| *Low* | *High* | 23 | 138.30 | 4 | 2.38 (0.84-6.74) | 2.30 (0.81-6.54) |
| *High* | *High* | 166 | 936.08 | 44 | 3.91 (2.47-6.18) | 3.75 (2.36-5.96) |
| **UNPROVOKED** |  |  |  |  |  |  |
| **D-dimer**  *(cutoff=215 ng/mL)* | **Thrombin Generation with high tissue factor, nAPCsr** | **N° patients**  539 | **Observation**  **years** | **N° recurrent VT**  115 | **Crude HR**  **(95% CI)** | **Sensitivity**  **Analysis**** |
| *Low* | *Low* | 97 | 589.90 | 8 | Reference | Reference |
| *High* | *Low* | 399 | 2237.67 | 87 | 2.85 (1.38-5.87) | 2.71 (1.31-5.60) |
| *Low* | *High* | 7 | 46.15 | 2 | 3.22 (0.68-15.17) | 2.82 (0.60-13.37) |
| *High* | *High* | 36 | 183.58 | 18 | 6.96 (3.02-16.02) | 7.10 (3.08-16.36) |
| **PROVOKED** |  |  |  |  |  |  |
| **D-dimer**  *(cutoff=215 ng/mL)* | **Thrombin Generation with high tissue factor, nAPCsr** | **N° patients**  1327 | **Observation**  **years** | **N° recurrent VT**  136 | **Crude HR**  **(95% CI)** | **Sensitivity**  **Analysis**** |
| *Low* | *Low* | 314 | 1981.52 | 23 | Reference | Reference |
| *High* | *Low* | 867 | 5104.72 | 85 | 1.43 (0.90-2.27) | 1.37 (0.86-2.18) |
| *Low* | *High* | 16 | 92.15 | 2 | 1.88 (0.44-7.98) | 1.85 (0.43-7.89) |
| *High* | *High* | 130 | 752.50 | 26 | 2.97 (1.69-5.20) | 2.76 (1.56-4.89) |

*D-dimer cut-off: low <=215, high >215; nAPCsr cutoff low <90° centile, high >=90° centile; ** sensitivity analysis: follow-up started at the date of blood sampling with adjustment for time between the date of discontinuation of anticoagulation therapy and blood sampling
